# Effects of *MDM2*, *MDM4* and *TP53* Codon 72 Polymorphisms on Cancer Risk in a Cohort Study of Carriers of *TP53* Germline Mutations

**DOI:** 10.1371/journal.pone.0010813

**Published:** 2010-05-26

**Authors:** Shenying Fang, Ralf Krahe, Guillermina Lozano, Younghun Han, Wei Chen, Sean M. Post, Baili Zhang, Charmaine D. Wilson, Linda L. Bachinski, Louise C. Strong, Christopher I. Amos

**Affiliations:** 1 Department of Epidemiology, University of Texas M.D. Anderson Cancer Center, Houston, Texas, United States of America; 2 Department of Genetics, University of Texas M.D. Anderson Cancer Center, Houston, Texas, United States of America; Health Canada, Canada

## Abstract

**Background:**

Previous studies have shown that MDM2 SNP309 and p53 codon 72 have modifier effects on germline P53 mutations, but those studies relied on case-only studies with small sample sizes. The impact of MDM4 polymorphism on tumor onset in germline mutation carriers has not previously been studied.

**Methodology/Principal Findings:**

We analyzed 213 p53 germline mutation carriers including 168(78.9%) affected with cancer and 174 who had genotypic data. We analyzed time to first cancer using Kaplan-Meier and Cox proportional hazards methods, comparing risks according to polymorphism genotypes. For *MDM2* SNP309, a significant difference of 9.0 years in the average age of cancer diagnosis was observed between GG/GT and TT carriers (18.6 versus 27.6 years, *P* = 0.0087). The hazards ratio was 1.58 (*P* = 0.03) comparing risks among individuals with GG/GT to risk among TT, but this effect was only significant in females (HR = 1.60, *P* = 0.02). Compared to other genotypes, *P53* codon 72 PP homozygotes had a 2.24 times (*P* = 0.03) higher rate for time to develop cancer. We observed a multiplicative joint effect of *MDM2* and *p*53 codon72 polymorphism on risk. The *MDM4* polymorphism had no significant effects.

**Conclusions/Significance:**

Our results suggest that the *MDM2* SNP309 G allele is associated with cancer risk in *p53* germline mutation carriers and accelerates time to cancer onset with a pronounced effect in females. A multiplicative joint effect exists between the *MDM2* SNP309 G allele and the *p53* codon 72 G allele in the risk of cancer development. Our results further define cancer risk in carriers of germline *p53* mutations.

## Introduction

p53 functions as a transcription factor and tumor suppressor, responding to cellular stresses such as DNA damage and oncogene activation. It modulates the transcription of genes that regulate cell cycle arrest, apoptosis, and senescence [1]. Aberrant function of p53 proteins is a frequent mechanism by which its inhibitory role in tumorigenesis is weakened, both in sporadic cancers, which often develop mutations of *p53*, and in individuals who inherit germline mutations. Mutations of *p53* account for the majority of families with Li-Fraumeni syndrome (LFS), an uncommon autosomal dominant cancer syndrome [2], [3]. Individuals with LFS are at an increased risk for a wide spectrum of neoplasms including breast, lung, brain, and adrenocortical cancers, and leukemias and sarcomas [4]–[6]. Unlike the dominant effect of germline *p53* mutation on cancer risk, germline *p53* polymorphisms exert more subtle effects on tumor onset or risk of cancer by modifying the function of *p53*. In particular, the codon 72 R/P polymorphism affects binding of p53 to p73 and has been associated with altered risk for many different cancers [7]–[9].

*MDM2* SNP309 (rs2279744; T/G) is located 309 base pairs downstream from intron 1 in the promoter of *MDM2*. The single nucleotide polymorphism (SNP) 309 T>G change has been found to enhance the affinity of the transcriptional activator Sp1, leading to increased levels of *MDM2*, and thereby weakening the *p53* pathway of tumor suppression [10]. In germline *p53* mutation carriers, SNP309 was reported to accelerate tumor onset and to be associated with the development of multiple primary tumors throughout the lifetime [10]–[12]}. The presence of the G allele was found to be highly related to earlier cancer diagnosis in LFS or Li-Fraumeni−like syndrome. The numbers of affected carriers of a germline *p53* mutation in three earlier published studies were small and analyses were restricted to include only individuals who had already developed cancer. Therefore, prior studies have limited generalizability for individuals at risk for cancer development due to inherited *p53* mutations who may not yet have developed cancer.

*MDM4 (MDMX)* is a negative regulator of *p53* and cooperates with MDM2 to inhibit p53 activity in cellular response to DNA damage. The human *MDM4* gene has been mapped to chromosome 1q32, a target for amplification in malignant gliomas [13]. While MDM4 inhibits p53 activity in early embryogenesis in animal models, MDM4 has a weak effect on p53 activity in many cell types [14]. Atwal et al(2009) reported that genetic variants in MDM4 led to an increased risk of early onset of human breast and ovarian cancers in unrelated individuals [15]. In another independent case-control study, a polymorphic variant in human MDM4 was only found to be associated with an accelerated age of onset of estrogen receptor negative breast cancer [16]. The impact of MDM4 on age of tumor onset in germline mutation carriers has not previously been investigated.

In this study, we investigated whether *MDM2* SNP309, *MDM4*, and *p53* codon 72 polymorphisms have any effect on risk for any type of cancer in carriers of a *p53* germline mutation. This is a long-term systematic follow-up study in which germline *p53* mutations and genetic polymorphisms were identified without respect to the cancer status in the family. This follow-up study with a larger sample size allowed us to characterize the cancer risks among carriers of germline *p53* mutations. We estimated hazard ratios by Kaplan-Meier methods and Cox regression to adjust for covariates and familial correlations by performing the robust sandwich estimate of Lin and Wei [17].

## Materials and Methods

### Study Population

The protocol and consent form is annually reviewed by the IRB at the University of Texas MD Anderson Cancer Center. No patient names are revealed in any reports or publications from this study. The present study population consisted of several cohorts of families that were identified through probands with early onset sarcoma or multiple cancers and that were found to carry *p53* germline mutations. One cohort comprises 107 kindreds identified through probands with soft-tissue sarcoma (STS) diagnosed before age 16 years during the years from 1944 to 1975 at The University of Texas M. D. Anderson Cancer Center (MDACC) who survived at least 3 years after diagnosis and had samples available for testing [2], [18], [19]. We identified 63 individuals in seven STS kindreds as carriers of a *p53* germline mutation. Another cohort included 71 families identified through probands who were diagnosed with osteosarcoma (OST) before age 20 years during the period from 1944 to 1982 at MDACC who had samples available for testing. We identified11 individuals in six OST kindreds who were carriers of a *p53* germline mutation. We also identified 2 carriers from two kindreds of probands with multiple primary malignant tumors and *p53* germline mutations. The remaining 137 carriers were identified from 59 LFS kindreds. Subjects were treated as a carrier of a *p53* germline mutation if they were shown by genetic testing to carry the mutation or if both a parent and offspring were demonstrated to carry the mutation, and thus positive mutation status could be inferred. We analyzed 213 carriers of a germline *p53* mutation in this study. Of the 213 individuals who could be inferred to have a *p53* mutation, samples were available for 132 individuals, but *MDM4* genotypes were missing for two of these individuals. A detailed description of the *p53* sequencing and genotyping procedures is provided in the supplemental materials(Text S1; Figure S1, Figure S2 and Figure S3; Table S1 and Table S2).

### Statistical Analysis

We first tested for differences in age at cancer diagnosis among the different genotype groups using a nonparametric Kruskal-Wallis test. Among the carriers of a germline *p53* mutation, we first performed a log-rank test for risk differences based on sex and mutation type, using the Kaplan-Meier product-limit method. Missing genotype data (n = 43, 44, and 42 for *MDM2*, *MDM4*, and *p53* codon 72, respectively) were imputed using Linkage software [20], and estimating population allele frequencies within each ethnicity. For this analysis, we estimated the likelihood of each genotype for individuals who had a *p53* mutation and at least one relative who had been genotyped for a *MDM2*, *MDM4*, or *p53* codon 72 polymorphism. The probability of a particular genotype was derived as the ratio of the likelihood for the family given that the mutation carrier had each particular genotype divided by the likelihood for the family. Genetic effects of *MDM2*, *MDM4*, and *p53* codon 72 polymorphisms were estimated by using a weighted Cox proportional hazard model, unadjusted or adjusted for sex, race, and birth year and weighted by the probability of each genotype (for the inferred data). We took into account the familial correlation in the model by calculating the robust variance. The time to onset was from birth to first cancer diagnosis, for those who had cancer, and the censoring time was from birth to last contact (fixed to December 31, 2001), death, or study termination, for those who had no cancer. All statistical analyses were conducted by using SAS 9.1 (SAS Institute, Cary, NC). A *P*-value<0.05 was considered statistically significant.

## Results

**Figure 1:**
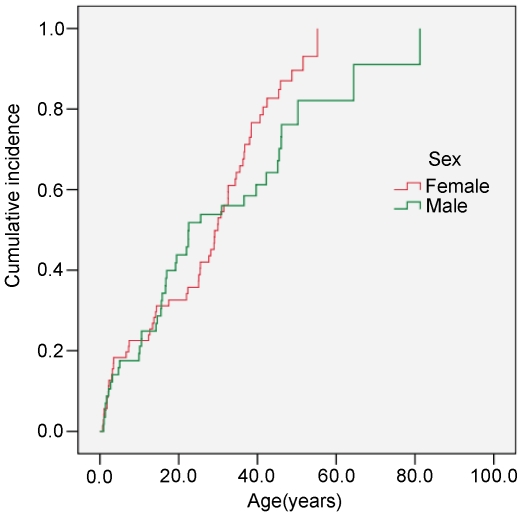
Kaplan-Meier estimated cumulative incidence for cancer in carriers of a *p53* germline mutation by sex. Observations included 101 males and 112 females (log-rank test *P* = 0.0057).

**Figure 2:**
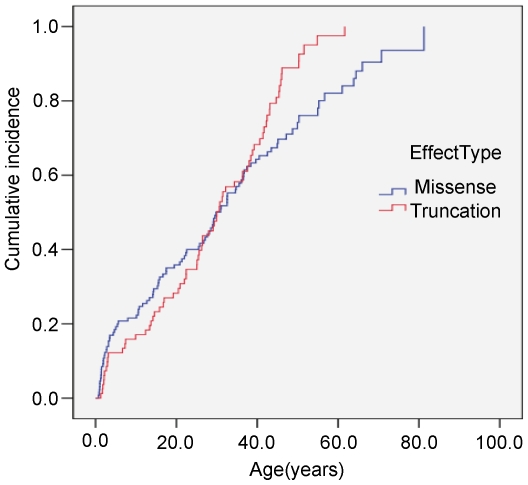
Kaplan-Meier estimated cumulative incidence for any cancer in carriers of a *p53* germline mutation by mutation type. Among these 213 carriers(one missing type of mutation), 130 carried a missense mutation and 82 a truncation mutation (deletion 1, nonsense 50, frame-shift 16, splice 15). Log-rank test *P* = 0.0900.

Of the 213 carriers with a *p53* germline mutation analyzed, 168 (78.9%) were affected with cancer, and the mean period from birth to cancer diagnosis or censoring was 27.9 years (SD = 18.2). Figure 1 illustrates the distribution of cancer occurrences by age and sex. Female mutation carriers were at higher risk than male carriers (log-rank test, *P* = 0.0057); the mean age of cancer diagnosis was 24 years in females and 26 years in males. No risk difference was detected between the two types of germline mutations, missense and truncating (log-rank test, *P* = 0.09) (Figure 2). Stratification analysis showed that the codon 72 polymorphisms in *cis* had no effect on age of tumor onset in carriers of dysfunctional missense mutations (*P* = 0.20) or truncating mutations (*P* = 0.78), and similarly in *trans* there was no significant effect when stratified by *p53* mutation type. Because there were missing genotypes for some carriers and a comparatively small sample of individual genotypes, hazard ratios (Table S3) estimated via the proportional Cox model restricted to only the raw genotype data have limited power. Table S4 shows that allelic distribution varied significantly among the different ethnicities for each polymorphism. The best genetic model for each SNP was determined by choosing the model with the lowest Akaike information criterion (AIC) value from among the general, dominant, recessive, and additive models (Table S5).

Comparing the age at diagnosis among those affected with cancer (table 1), a significant average difference of 9.0 years was observed for the carriers of a G allele for the *MDM2* SNP309 polymorphism compared to TT carriers (18.6 versus 27.6 years, *P* = 0.0087). When analyzing time to onset including affected and unaffected individuals who were genotyped we did not observe a significant difference among genotypes for the *MDM2* SNP309 (*P* = 0.5557) (Figure S4). Stratification analysis showed that G allele carriers had a worse survival than TT homozygotes among females(Log-Rank test P = 0.1483, Wilcoxon test P = 0.0950)(Figure S5), but those two genotype groups among males had the same survival distributions over time (P>0.1 for both Log-Rank test and Wilcoxon test)(Figure S6). When including the imputed data, a trend towards significance was noted for the univariable analysis of the *MDM2* G allele (*P* = 0.0764 unadjusted and *P* = 0.1067 adjusted analysis) (Table 2), but carriers of a G allele had a 1.58 fold increased risk for cancer after adjusting for sex, race, birth year, and effects from other polymorphisms in multivariable analysis (*P* = 0.0313) (Table 3). Including an interaction term between *MDM2* SNP309 polymorphism and sex revealed that the G allele was a risk allele among females (*P* = 0.02) but not among males (*P* = 0.1936). To limit possible effects of referral bias, further multivariable analysis was performed among carriers of a *p53* germline mutation after excluding the probands and yielded similar results. Carriers of the *MDM2* G allele had a high risk among all relatives (*P* = 0.0117) or female relatives (*P* = 0.0089), but no significant effect was noted among male relatives (*P* = 0.1315) (Table S6).

**Table 1:** Mean age of tumor diagnosis in affected carriers of a *p53* germline mutation by *p53* polymorphism.

Polymorphism	Subcategory	N (%)	Mean age, years (SD)	*P*-value*
*MDM2* SNP309	GG	15(14.7)	23.5(16.9)	0.0119
	GT	38(37.3)	16.7(13.9)	
	TT	49(48.0)	27.6(18.1)	
	GG+GT	53(52.0)	18.6(14.9)	0.0087
*MDM4*	AA	16(16.0)	22.2(16.6)	0.9680
	AG	38(38.0)	21.7(15.3)	
	GG	46(46.0)	23.2(18.7)	
*p53* codon 72	PP	7(6.9)	18.5(11.2)	0.3463
	RP	47(46.1)	25.0(16.6)	
	RR	48(47.0)	21.8(18.0)	
	RP+RR	95(93.1)	23.4(17.3)	0.5828

* Kruskal-Wallis test.

**Table 2:** Univariable analysis of *MDM2*, *MDM4*, and *p53* codon72 polymorphisms on age of tumor diagnosis using raw plus imputed genotype data among carriers of a *p53* germline mutation*.

		Unadjusted		Adjusted**	
Variable	Subcategory	Hazard Ratio	*P*-value	Hazard Ratio	*P*-value
*MDM2* (G dominant, n = 175)	GG/GT = 1,TT = 0	1.45(0.96–2.19)	0.0764	1.40(0.93–2.10)	0.1067
*MDM4* (G dominant,n = 174)	AG/GG = 1,AA = 0	1.43 (0.93–2.22)	0.1054	1.73(0.81–3.72)	0.1584
*p53* codon 72(P rec, n = 174)	PP = 1,PR/RR = 0	2.22(1.27–3.89)	0.0052	2.03(1.06–3.89)	0.0327
Mutation type(n = 175)	Missense = 1,Truncating = 0	0.76(0.46–1.27)	0.2989		
Missense-*Cis*72(n = 71)	P = 1,R = 0	1.41(0.83–2.41)	0.2026		
Missense-*Trans*72(n = 71)	P = 1,R = 0	1.35(0.71–2.57)	0.3546		
Truncating- *Cis*72(n = 47)	P = 1,R = 0	0.89(0.39–2.04)	0.7810		
Truncating- *Trans*72(n = 47)	P = 1,R = 0	0.72(0.44–1.19)	0.2040		
Sex(n = 213)	Female = 1	1.56(1.15–2.11)	0.0039		
Race(n = 213)	Black	1.16(0.65–2.05)	0.2081		
	Other	1.50(0.95–2.35)			
Birth year(n = 213)		1.04(1.03–1.05)	<0.0001		

*Cox regression model.

**Adjusted for sex, race, and birth year in Cox regression model.

**Table 3:** Multivariable analysis of hazard ratios for *MDM2*, *MDM4*, and *p53* codon 72 polymorphisms on age of tumor diagnosis among carriers of a *p53* germline mutation.

		All(n = 174)*		Male(n = 83)**		Female(n = 91)**	
Variable	Subcategory	Hazard Ratio	*P*-value	Hazard Ratio	*P*-value	Hazard Ratio	*P*-value
*MDM2*	GG/GT	**1.58(1.04–2.26)**	**0.0313**	1.47(0.82–2.60)	0.1936	**1.60(1.08–2.36)**	**0.0200**
	TT	1.00		1.00		1.00	
*MDM4*	AG/GG	1.93(0.95–3.93)	0.0712	1.74(0.93–3.25)	0.0842	2.39(0.97–5.88)	0.0589
	AA	1.00		1.00		1.00	
*p53* codon 72	PP	**2.24(1.09–4.60)**	**0.0287**	**4.27(2.56–7.11)**	**<0.0001**	1.33(0.59–3.01)	0.4864
	PR/RR	1.00		1.00		1.00	
Sex	Female	1.39(0.94–2.04)	0.0970	-	**-**	-	-
	Male	1.00		-		-	
Race	Black	2.28(1.25–4.15)	**0.0071**	2.17(1.14–4.13)	**0.0190**	2.17(1.14–4.13)	**0.0190**
	Others	0.99(0.62–1.56)	0.9523	0.99(0.64–1.53)	0.9447	0.99(0.64–1.53)	0.9447
	White	1.00		1.00		1.00	
Birth year		1.04(1.03–1.06)	**<0.0001**	1.04(1.03–1.06)	**<0.0001**	1.04(1.03–1.06)	**<0.0001**

*Adjusted for gender, race and birth year.

**Adjusted for race and birth year.

For *MDM4*, we identified no significant difference in the average ages of first cancer diagnosis between AA, AG, and GG groups (*P* = 0.9680) (Table 1). The log-rank test result shows no difference in risk of cancer among these three genotypes (*P* = 0.6646) or between the AG/GG and AA groups (*P* = 0.3770) (Figure S7). The *MDM4* polymorphism did not have a significant effect on risk of developing cancer because it was not significant in unadjusted (*P* = 0.1054) or adjusted univariable analysis (*P* = 0.1584) (Table 2) or multivariable analysis (*P* = 0.0712) of raw plus inferred genotype data (Table 3). No significant difference was found for *MDM4* polymorphism when probands were excluded from the analysis (*P* = 0.0752) (Table S6). While no significant effects were observed in this study, studies in a larger collection of families are needed to resolve whether *MDM4* has any effect on risk for cancer among carriers of a *p53* mutation.

For the *p53* codon 72 polymorphism, only seven mutation carriers had the PP genotype. A difference of 4.9 years in mean age at cancer diagnosis was detected between PP and RP/RR groups, but the difference was not significant in univariate analyses (18.5 years versus 23.4, *P* = 0.5828) (Table 1). There was no significant difference in survival curves among PP, PR, and RR groups (*P* = 0.0955) when the joint distributions of time to diagnosis among all genotypes were contrasted, but the time to diagnosis differed significantly between PP and either PR or RR genotypes (*P* = 0.0447) according to the log-rank test on genotyped data (Figure S8). In the full sample, including inferred data, the codon 72 P allele was a risk allele for cancer in the unadjusted univariable analysis (*P* = 0.0052), adjusted univariable analysis (*P* = 0.0327) (Table 2), and multivariable analysis after adjusting for covariates and other SNPs (HR = 2.24, *P* = 0.0287) (Table 3). Further analysis showed that the PP genotype had a significant recessive effect on cancer development among males (*P*<0.0001), but not among females (*P* = 0.4864). The hazard ratios increased and *P*-values became smaller if multivariable analysis excluded probands (*P*<0.0001), and the *P*-value was significant among both males (*P*<0.0001) and females (*P*<0.0001) (Table S6).

Because both *MDM2* SNP309 and *p53* codon 72 polymorphism can attenuate the inhibitory role of p53 in tumorigenesis [21], we examined the joint effect of *MDM2* and *p*53 codon72 polymorphism (Table 4). Compared with the reference group carrying no risk genotype at either locus (i.e., *MDM2* TT and *p53* codon 72 PR/RR), those with a risk genotype on one of the loci, *MDM2* (*MDM2* GG/GT and *p53* codon 72 PR/RR) were 1.54 times more likely to have cancer (*P* = 0.0319), and the highest hazard ratio of 3.25 was observed for those carriers with risk genotypes at both loci (*P* = 0.0367); this hazard ratio is close to the product of hazard ratios for the main effects of risk genotype at each locus (1.54×2.36 = 3.63), suggesting that the two SNPs together have a multiplicative joint effect.

**Table 4:** Risk of cancer associated with joint effect of *MDM2* and *p53* codon 72 polymorphisms.

*MDM2*	*p53* Codon 72	Hazard Ratio*	P>ChiSq
TT	PR/RR	1.00	
	PP	2.36(0.85–6.56)	0.0994
GT/GG	PR/RR	1.54(1.04–2.29)	0.0319
	PP	3.25(1.08–9.84)	0.0367

*Adjusted for sex, race, birth year, and *MDM4*.

It is noteworthy that carrier birth year was a significant covariate in both univariable (Table 2) and multivariable analysis (Table 3). In carriers of a *p53* germline mutation, each subsequent date of birth increased the cancer risk by 3% (*P*<0.0001). This trend was observed for both men and women (Table 3). The findings suggest genetic anticipation in later birth cohorts or effects from unmeasured environmental factors that have an increasing effect on risk over time.

## Discussion

In this study, we evaluated whether specific genetic polymorphisms have any impact on risk of cancer in carriers of a *p53* germline mutation. Among *p53* carriers, cancer risk was significantly higher in females than in males, but no difference in cancer risk was found between missense and truncating mutation groups. Our results demonstrate that *MDM2* SNP309 and *p53* codon 72 polymorphisms have strong genetic effects in carriers of a *p53* germline mutation. Cancer diagnosed in affected carriers with *MDM2* GG/GT was on average 9 years earlier than that in affected carriers carrying the TT genotype. Although *MDM2* SNP309 was not a significant cancer risk factor via the log-rank test or in univariable analysis, it was linked to a 1.58 times greater likelihood of developing cancer than TT homozygosity after adjusting for other confounders. A significant SNP309 effect was observed in women but not in men. Patients with *p53* 72P developed cancer 5 years earlier than individuals with RP/RR genotypes, but the difference was not significant. In Cox regression analysis, the *p53* codon 72 PP genotype carried a significantly higher risk of developing cancers. Our results indicate that a multiplicative joint effect exists between the *MDM2* and the *p53* codon 72 polymorphism. However, no significant effects were observed between *MDM4* and cancer risk in germline mutation carriers.

Bond et al. (2004) analyzed 88 affected mutation carriers and found that the median age of tumor onset for those who carried GG/GT (18 years) was 9.0 years earlier than that for those carrying TT (27 years) (*P* = 0.031) [10]. The present study is a continuing follow-up cohort including some cancer cases studied by Bond. However, our study is more accurate because it has more samples and includes all p53 carriers, not just those who had cancer. Bougeard et al. (2006) showed that, among 61 French carriers of a germline *p53* mutation (41 affected with cancer), the mean age of tumor onset in those with *MDM2* SNP309 GG/GT (19.6 years) was significantly younger than in those with *MDM2* TT (29.9 years) (*P*<0.05) [11]. Marcel et al. (2009) demonstrated that, in a group of 32 cancer-affected Brazilian patients with LFS or Li-Fraumeni−like syndrome and a germline *p53* mutation, the presence of a G allele was associated with a 12.5-year earlier diagnosis (GG/GT 26.3 years versus TT 38.8; *P* = 0.06) [12]. So far all previous studies consistently show that *MDM2* SNP309 can accelerate tumor formation in carriers of a germline *p53* mutation. In the present study, comparison of mean age of tumor diagnosis between affected carriers with different *MDM2* SNP309 genotypes revealed a significant difference, but the genotype did not significantly affect the hazard for cancer development among all carriers. When we adjusted for confounders, the MDM2 SNP309 effect became significant overall and we observed a 58% higher cancer risk in the G allele carriers compared with TT homozygotes. We also observed a higher risk from the MDM2 SNP309 genotypes in females, compared to males. The more pronounced effect in females that we observed may relate to biological regulation of MDM2 by estrogen. *MDM2* SNP309 is located in a region of the *MDM2* promoter regulated by hormonal signaling pathways. The G allele was demonstrated to enhance the affinity of a co-transcriptional activator of multiple hormone receptors, for example ER or Sp1. Bond et al. (2006) showed that this polymorphism accelerated tumor formation in a gender-specific fashion, and depended upon estrogen signaling [22]. This finding suggested a genotype-dependent role for clinical manipulation of hormone level in cancer prevention and treatment. Interestingly, Bond et al. had a similar finding in 162 patients with diffuse large B-cell lymphoma, where the G allele contributed to earlier tumor onset only among females, but not among males [22].

Bougeard et al. showed that the presence of the *p53* 72 R allele accelerated tumor onset by 12.6 years in carriers of a germline *p53* mutation (*P*<0.05) [11]. Marcel et al. reported that the R allele reduced age at cancer diagnosis by almost 8 years in individuals with LFS or Li-Fraumeni−like syndrome, although the difference was not significant (*P* = 0.22) [12]. Our findings that the PP genotype increased risk after adjusting for cohort effects were in conflict with those of the previous two studies, but were consistent with the report of Martin et al. that the P72 allele was a risk factor for breast cancer in 84 carriers with *BRCA1* mutation [8].. Dumont et al. reported that the *p53* 72R variant was 5- to 10-times more likely to induce programmed cell death than the 72P variant, and the authors suggested that the low apoptotic potential of the 72P variant might account for increased predisposition to cancer development in carriers of the 72P variant [23].

In conclusion, our study confirms that the *MDM2* SNP309 G allele is associated with cancer risk in carriers of a *p53* germline mutation and that it accelerates tumor formation with a pronounced effect in females. Our results also suggest that *p53* codon 72 PP homozygosity is a risk factor for cancer. We found a joint multiplicative effect of *MDM2* SNP309 G allele and *p53* codon 72 PP homozygosity. Our results provide insights that SNPs further modify the risk for cancer development in individuals with *p53* mutations. In addition, given the high prevalence of *p53* mutations in sporadic cancers, our findings may generalize to a broader set of cancers.

## Supporting Information

Text S1 Supplemental methods.
(0.04 MB DOC)

Figure S1 Sequencing representation of a wild-type and a mutation and/or polymorphism.
(0.76 MB TIF)

Figure S2 Representative programs of all three possible genotypes for SNPs TP53 P72R.
(0.54 MB TIF)

Figure S3 Representative programs of all three possible genotypes for MDM2 SNP309.
(0.53 MB TIF)

Figure S4 Proportion of subjects who were cancer free by MDM2 SNP309 polymorphism at different ages. Log-rank test among GG, GT, and TT, P = 0.5557, and between GG+GT and TT, P = 0.3654.
(0.60 MB TIF)

Figure S5 Proportion of female subjects who were cancer free by MDM2 SNP309 polymorphism at different ages. Log-rank test among GG,GT and TT, P = 0.1864, Wilcoxon test P = 0.2414; Log-rank test between GG+GT and TT, P = 0.1483, Wilcoxon test P = 0.0950.
(0.60 MB TIF)

Figure S6 Proportion of male subjects who were cancer free by MDM2 SNP309 polymorphism at different ages. Log-rank test among GG,GT and TT, P = 0.9906, Wilcoxon test P = 0.5885; Log-rank test between GG+GT and TT, P = 0.9881, Wilcoxon test P = 0.9001.
(0.55 MB TIF)

Figure S7 Proportion of subjects who were cancer free by MDM4 polymorphism at different ages. Log-rank test among AA, AG, and GG, P = 0.6646, and between AA and AG+GG, P = 0.3770.
(0.58 MB TIF)

Figure S8 Proportion of subjects who were cancer free by p53 codon 72 polymorphism at different ages. Log-rank test among PP, PR, and RR, P = 0.0955, and between PP and PR+RR, P = 0.0447.
(0.60 MB TIF)

Table S1 Detection of germline p53 mutations.
(0.06 MB DOC)

Table S2 Primer sequences for genotyping assays.
(0.03 MB DOC)

Table S3 Univarible and multivariable analyses of MDM2, MDM4, and p53 codon 72 polymorphisms on age of tumor diagnosis using raw genotype data from carriers of a p53 germline mutation.
(0.04 MB DOC)

Table S4 Distribution of allele frequencies by ethnicity.
(0.03 MB DOC)

Table S5 Genetic model selection using AIC in univariable analysis of MDM2, MDM4, and p53 codon 72 polymorphisms on age of tumor diagnosis using raw plus imputed genotype data among carriers of a p53 germline mutation.
(0.05 MB DOC)

Table S6 Multivariable analysis of hazard ratio for MDM2, MDM4, and p53 codon 72 polymorphisms on age of tumor diagnosis among carriers of a p53 germline mutation, probands excluded (n = 126).
(0.04 MB DOC)
