# Supplementary material for: Effects of MDM2, MDM4 and TP53 Codon 72 Polymorphisms on Cancer Risk in a Cohort Study of Carriers of TP53 Germline Mutations
Source: PLoS One. 2010 May 26;5(5):e10813. doi: 10.1371/journal.pone.0010813 (PMC2877078; doi:10.1371/journal.pone.0010813)
Supplement: Text S1 — Supplemental methods. (0.04 MB DOC) [file pone.0010813.s001.doc]

**Supplemental methods: Mutation Detection and Polymorphism Genotyping**

Genomic DNA from peripheral blood lymphocytes was extracted by using either a modification of a protein salting-out protocol [1] or standardized DNA extraction via Autopure LS technology(Qiagen, Inc., Valencia, CA) in the Biospecimen Extraction Facility at MDACC. Samples were analyzed for *p53* variations in the coding regions by direct sequencing of PCR products. Intronic primers were designed for PCR amplification of each coding exon (2-11) (Table S1). PCR reactions contained 100 ng of genomic DNA, 0.5 μl of each primer (10 μM), 0.25 μl of Amplitaq Gold ® DNA Polymerase (5U/ μl), 5 μl of 10× PCR Buffer II, 5 μl of MgCl_2_ solution (25 mM), and 5 μl of dNTP (2.5 mM) with a total volume of 50 μl. The PCR thermal cycles (Biorad DNA Engine Peltier Thermal Cycler, Bio-Rad Laboratories, Hercules, CA) used to amplify the exons consisted of an initial cycle of 95°C for 7 minutes, followed by 35 cycles at 95°C for 40 sec, 62°C for 40 sec, 72°C for 1 minute, and a final extension at 72°C for 10 minutes. For exons 3 and 4, the annealing temperature was 58°C, and for exon 11 the annealing temperature was 55°C. PCR products were visualized by electrophoresis in 1.5% agarose with 0.1 μg/ml ethidium bromide. Subsequently, PCR products were purified and sequenced using the same PCR primers in an ABI capillary DNA sequencer and ABI capillary genetic analyzer(Applied Biosystems, Inc., Foster City, CA) in the DNA Analysis Facility at MDACC. Sequence chromatograms were assessed automatically and visually, and sequence alterations were confirmed from a new PCR reaction and by DNA sequence analysis in the opposite direction (Figure S1). For identification of *p*53 genomic DNA nucleotide numbering and codon numbering systems sequence files were compared to the those from Genbank(Accesions X54156 and X02469). Mutations (1 missing) were classified as truncating if they resulted from nonsense (n=50), large deletion (n=1) or insertion (n=0), frameshift(n=16), or splice-site mutation (n=15). Missense mutations (n=130) included single-site mutations or in-frame deletions/insertions that altered the encoded amino acid.

##### Polymorphisms of p53 P72R and MDM2 SNP309 (rs2279744) were genotyped by Qiagen Pyrosequencing according to the manufacturer’s instructions. PCR primers and sequencing primers were designed using the Pyrosequencing PSQ Assay Design software. PCR was performed in a 25-μl reaction containing Qiagen HotStart Taq master mix and 10 ng of genomic DNA. For amplification, we used a three-primer universal primer protocol as previously described[2,3]. Briefly, gene-specific primers containing a universal tail were used at a ratio of 1:9 (gene-specific primer: universal primer) with the biotinylated universal primer to generate biotinylated products for Pyrosequencing (Table S2). Amplification was carried out as follows: initial denaturation at 95^o^C for 5 minutes, followed by 50 cycles of 95^o^C for 30 seconds, 58^o^C (TP53 R72P) or 55^o^C (MDM2 SNP309) for 45 seconds, and 72^o^C for 45 seconds, and a final extension of 72^o^C for 7 minutes. Subsequent Pyrosequencing was performed on a PSQ96HS system (Qiagen) according to manufacturer’s instructions using the single-strand binding protein protocol (PyroGold). Genotypes were scored using the Q-CpG software (Qiagen). Representative pyrograms of all three possible genotypes for TP53 P72R and MDM2 SNP309 are provided in Figure S2 and Figure S3, respectively.

The SNP in intron 10 of the *MDM4* gene (rs1563828) was genotyped using the Taqman SNP genotyping assay from (Applied Biosystems, assay number C_9493064_10). A limited number of *MDM4* intron 10 genotypes were confirmed by PCR amplification using primers 5′- CACAGATTAGTATTAATGGAGGAC-3’(forward) and 5’-CCTTCTTATCCTTCCTCCACCAAC-3’(reverse). PCR products were separated by electrophoresis, then subjected to DNA purification and then sequenced by using the forward and reverse primers. Sequences were analyzed using the Chromas software.

Supplemental reference:

1. Miller SA, Dykes DD, Polesky HF (1988) A Simple Salting Out Procedure for Extracting Dna from Human Nucleated Cells. Nucleic Acids Research 16: 1215.

2. Bachinski LL, Udd B, Meola G, Sansone V, Bassez G, et al. (2003) Confirmation of the type 2 myotonic dystrophy (CCTG)n expansion mutation in patients with proximal myotonic myopathy/proximal myotonic dystrophy of different European origins: a single shared haplotype indicates an ancestral founder effect. Am J Hum Genet 73: 835-848.

3. Colella S, Shen L, Baggerly KA, Issa JP, Krahe R (2003) Sensitive and quantitative universal Pyrosequencing methylation analysis of CpG sites. Biotechniques 35: 146-150.
