# Supplementary figures and images for: Effects of MDM2, MDM4 and TP53 Codon 72 Polymorphisms on Cancer Risk in a Cohort Study of Carriers of TP53 Germline Mutations
Source: PLoS One. 2010 May 26;5(5):e10813. doi: 10.1371/journal.pone.0010813 (PMC2877078; doi:10.1371/journal.pone.0010813)

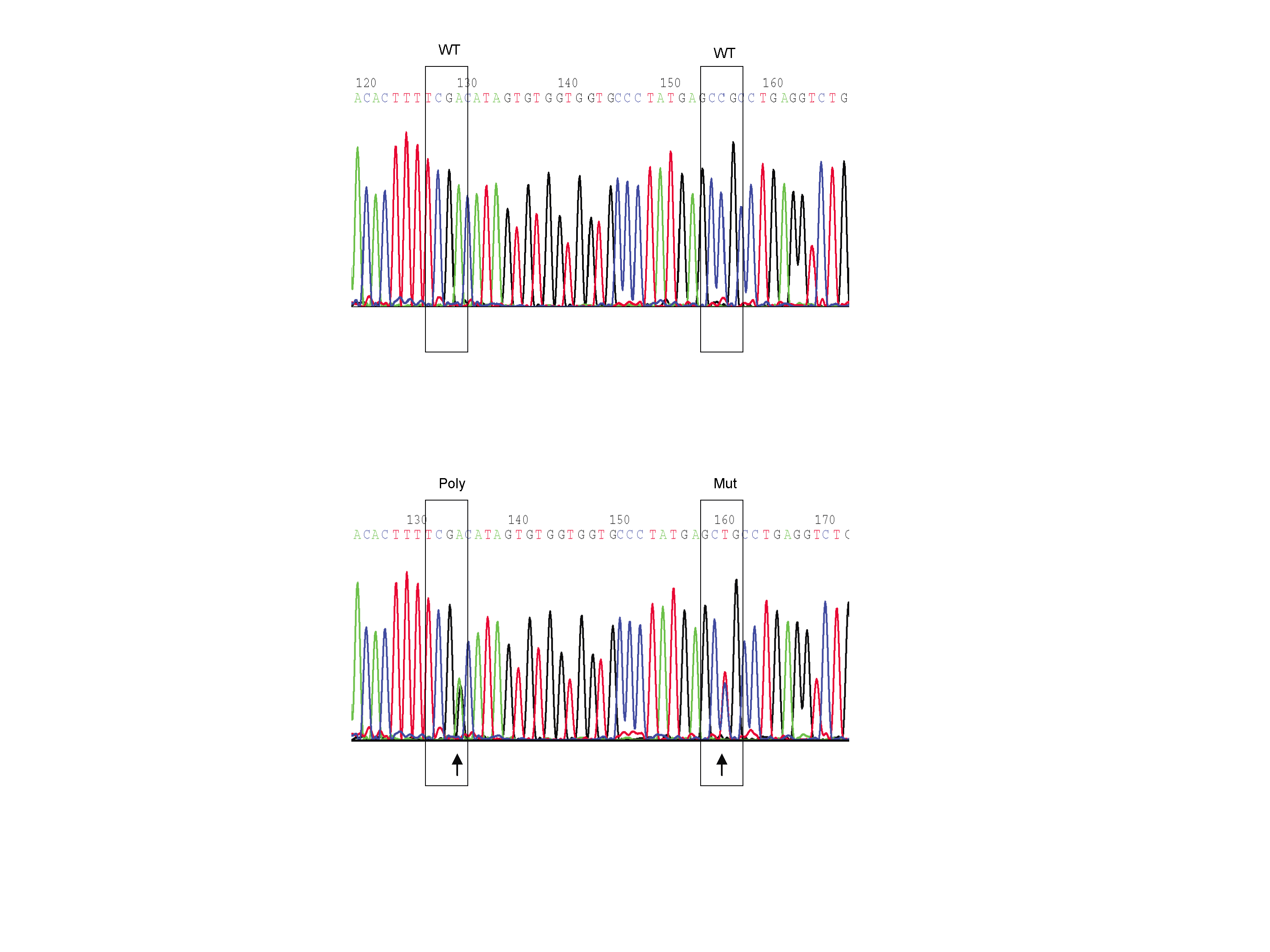

Supplement: Figure S1 — Sequencing representation of a wild-type and a mutation and/or polymorphism. (0.76 MB TIF) [file pone.0010813.s002.tif]

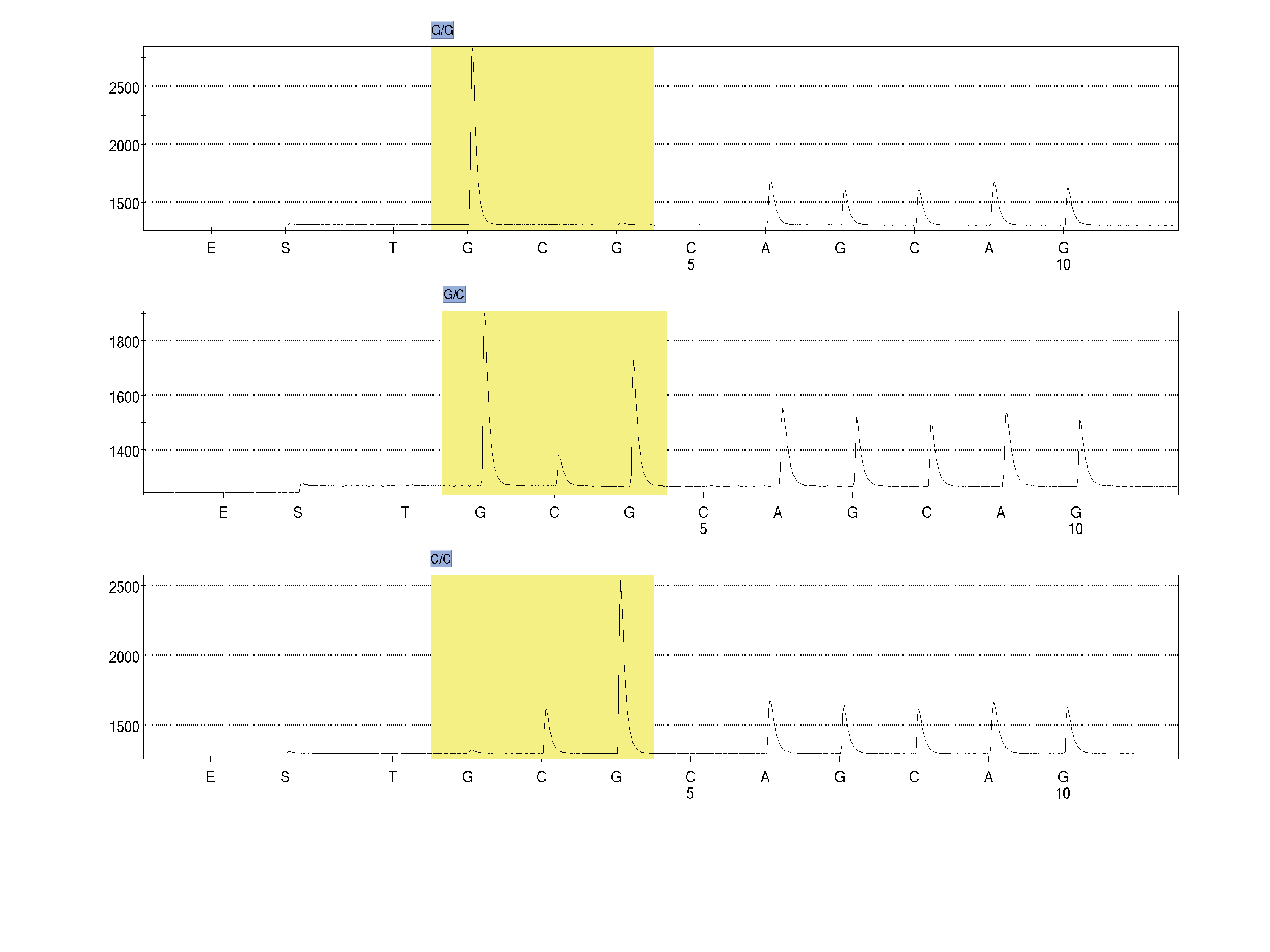

Supplement: Figure S2 — Representative programs of all three possible genotypes for SNPs TP53 P72R. (0.54 MB TIF) [file pone.0010813.s003.tif]

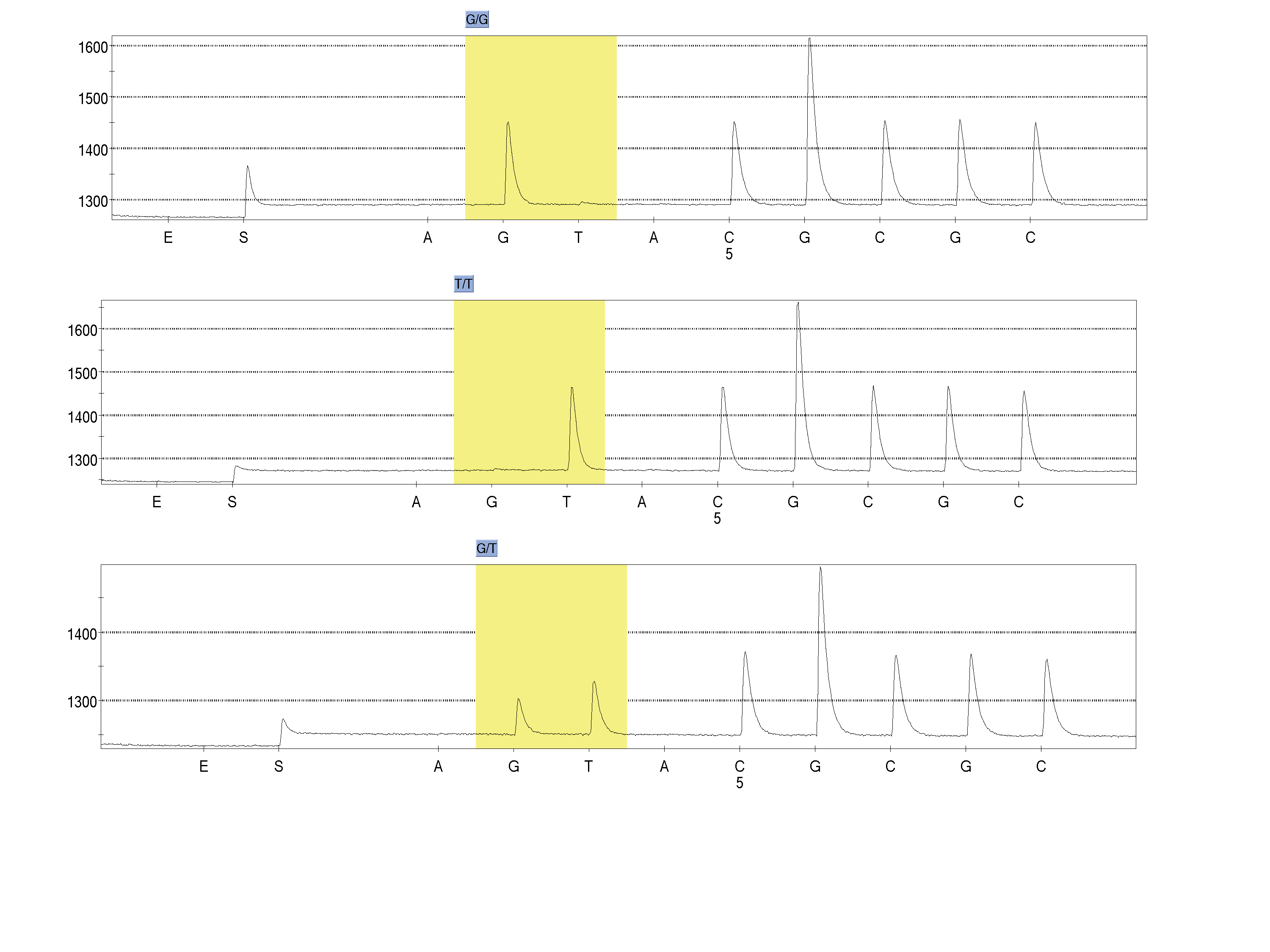

Supplement: Figure S3 — Representative programs of all three possible genotypes for MDM2 SNP309. (0.53 MB TIF) [file pone.0010813.s004.tif]

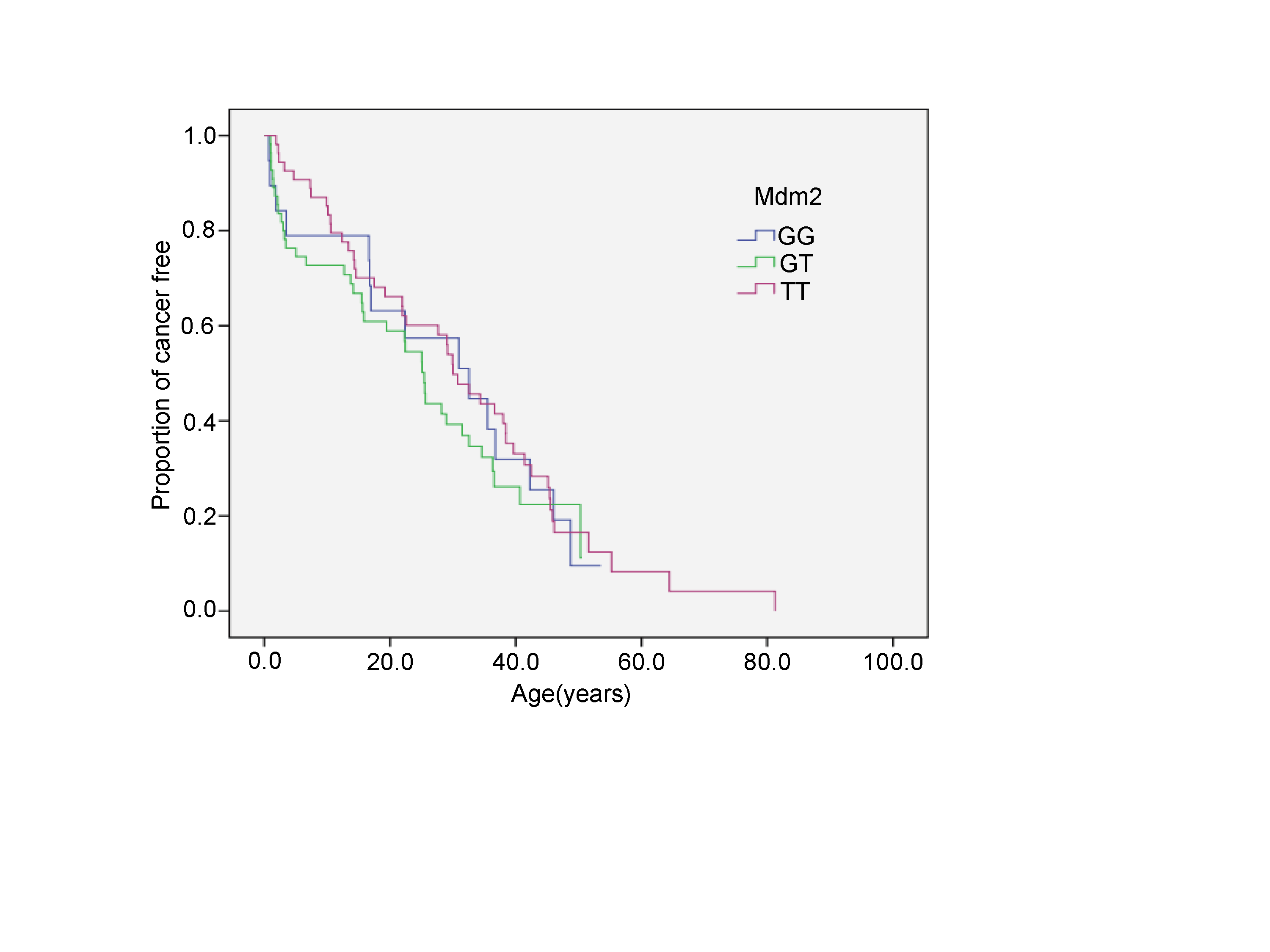

Supplement: Figure S4 — Proportion of subjects who were cancer free by MDM2 SNP309 polymorphism at different ages. Log-rank test among GG, GT, and TT, P = 0.5557, and between GG+GT and TT, P = 0.3654. (0.60 MB TIF) [file pone.0010813.s005.tif]

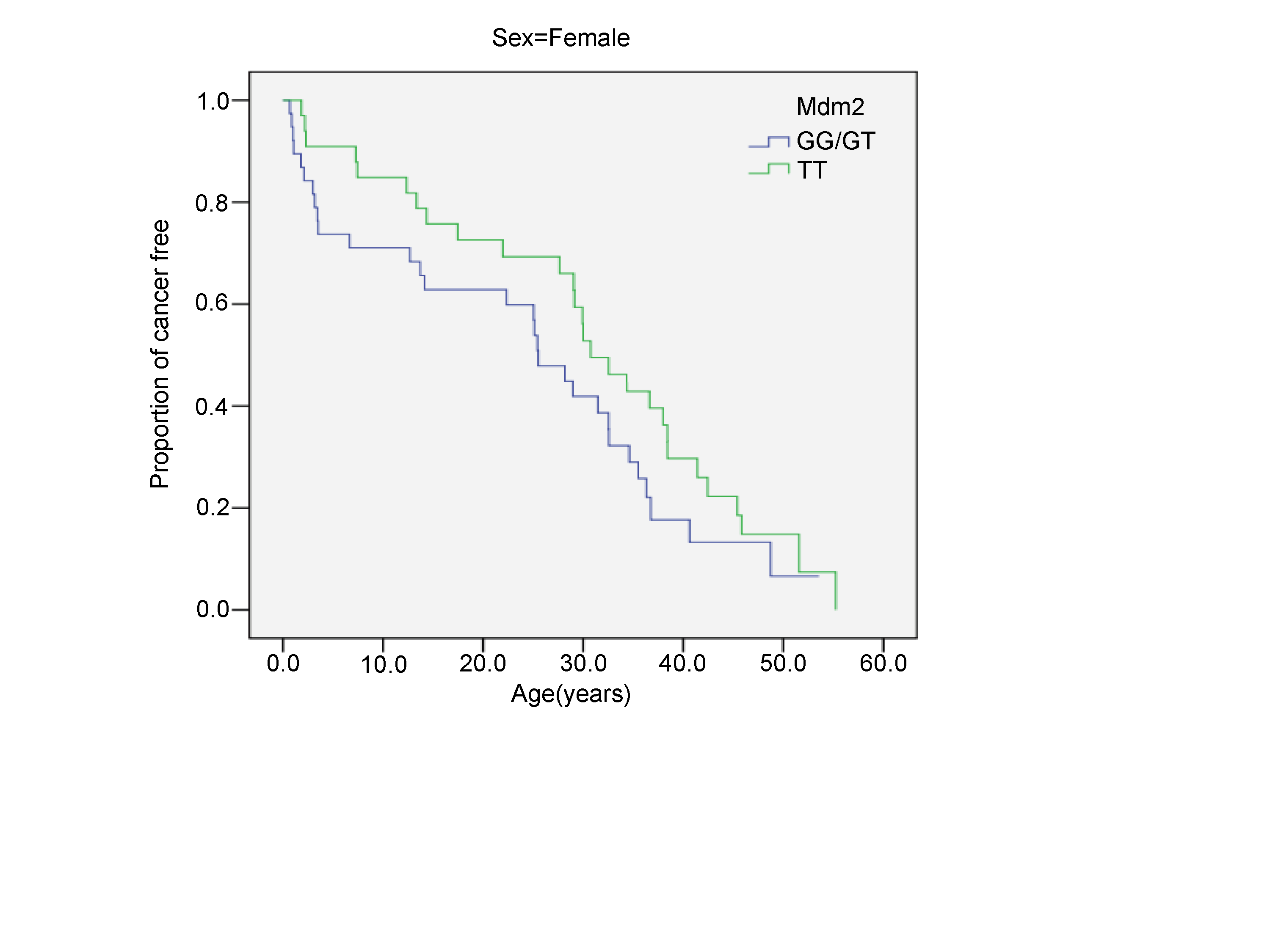

Supplement: Figure S5 — Proportion of female subjects who were cancer free by MDM2 SNP309 polymorphism at different ages. Log-rank test among GG,GT and TT, P = 0.1864, Wilcoxon test P = 0.2414; Log-rank test between GG+GT and TT, P = 0.1483, Wilcoxon test P = 0.0950. (0.60 MB TIF) [file pone.0010813.s006.tif]

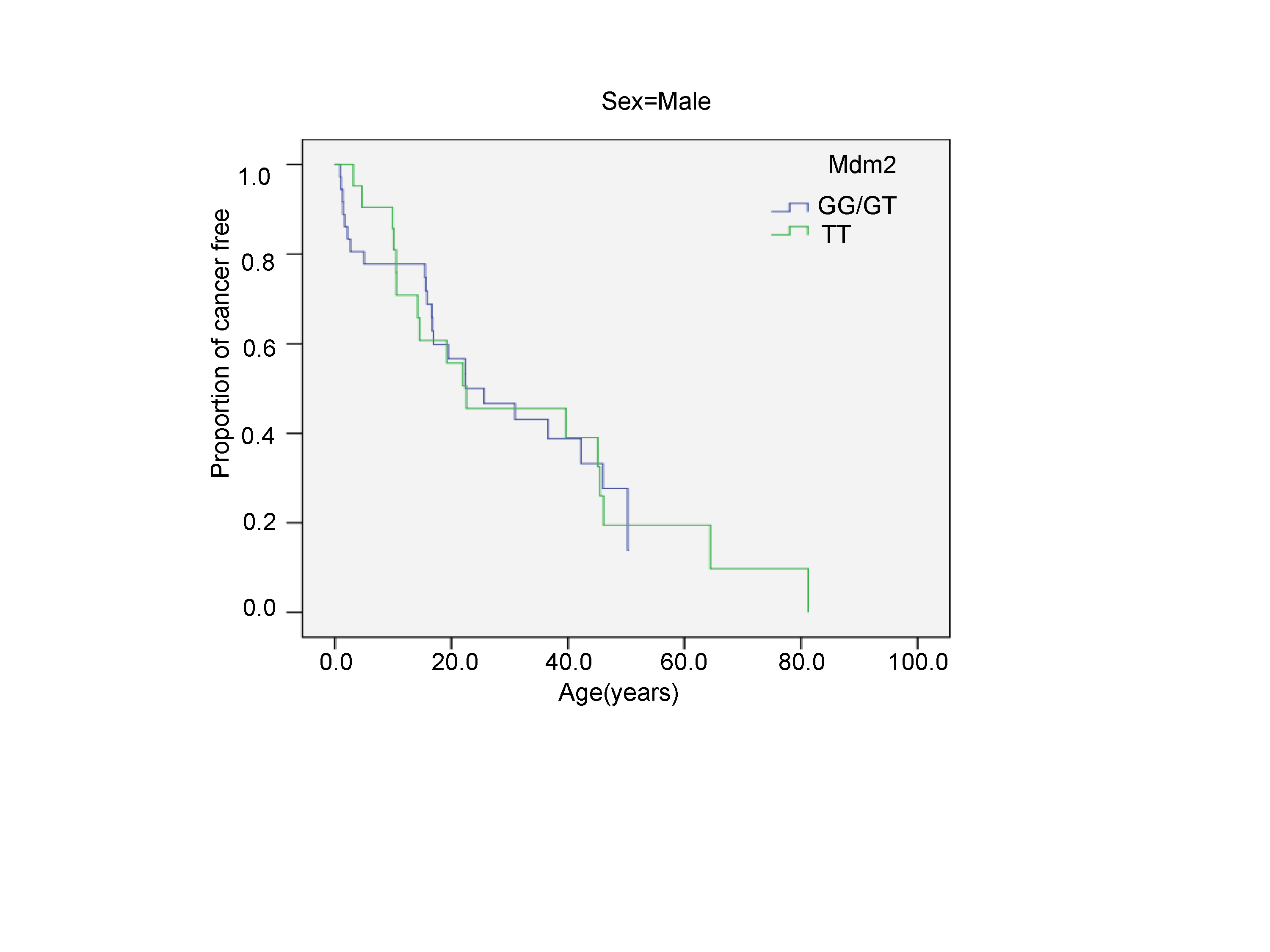

Supplement: Figure S6 — Proportion of male subjects who were cancer free by MDM2 SNP309 polymorphism at different ages. Log-rank test among GG,GT and TT, P = 0.9906, Wilcoxon test P = 0.5885; Log-rank test between GG+GT and TT, P = 0.9881, Wilcoxon test P = 0.9001. (0.55 MB TIF) [file pone.0010813.s007.tif]

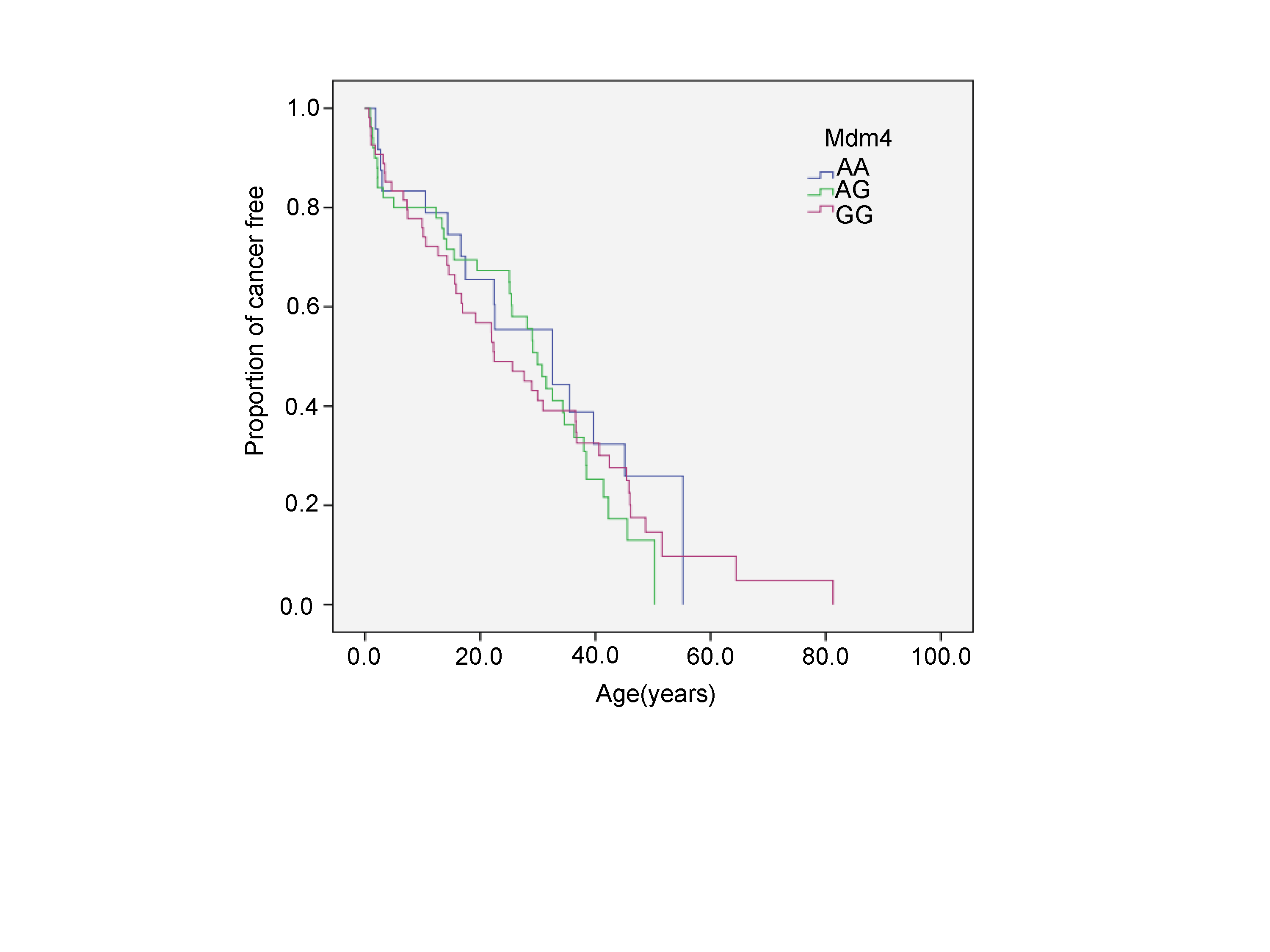

Supplement: Figure S7 — Proportion of subjects who were cancer free by MDM4 polymorphism at different ages. Log-rank test among AA, AG, and GG, P = 0.6646, and between AA and AG+GG, P = 0.3770. (0.58 MB TIF) [file pone.0010813.s008.tif]

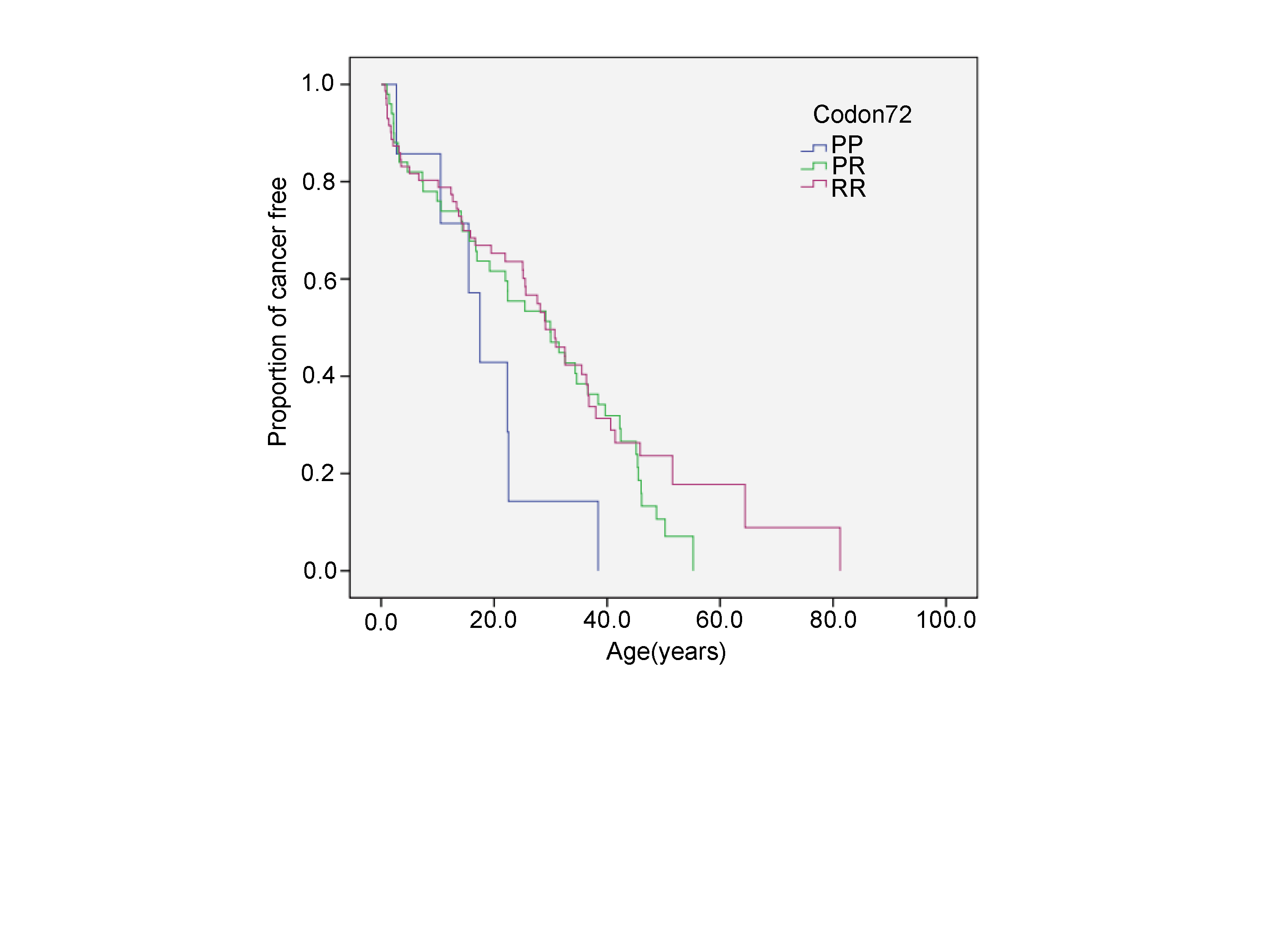

Supplement: Figure S8 — Proportion of subjects who were cancer free by p53 codon 72 polymorphism at different ages. Log-rank test among PP, PR, and RR, P = 0.0955, and between PP and PR+RR, P = 0.0447. (0.60 MB TIF) [file pone.0010813.s009.tif]
