# Supplementary material for: Effects of MDM2, MDM4 and TP53 Codon 72 Polymorphisms on Cancer Risk in a Cohort Study of Carriers of TP53 Germline Mutations
Source: PLoS One. 2010 May 26;5(5):e10813. doi: 10.1371/journal.pone.0010813 (PMC2877078; doi:10.1371/journal.pone.0010813)
Supplement: Table S1 — Detection of germline p53 mutations. (0.06 MB DOC) [file pone.0010813.s010.doc]

| **Exon** | **Fragment Size (bp)** | **Primer Label** | **Primer Sequence(5’ to 3’)** | **Primer Position Numbers** | **p53 Exon Position Numbers** |
| --- | --- | --- | --- | --- | --- |
| 2 | 220 | p53 × 2Fa | GGC ATG GTG TTG GGG GAG GGG | 11594-11614 | **E2** 11689-11790 |
|  |  | p53 × 2Ra | TGG GGT CGG GGT GGT GGC CTG | 11833-11813 |  |
| 3 | 214 | Ex3F | GAG ACC TGT GGG AAG CGA AAA | 11,851-11,871 | **E3** 11906-11927 |
|  |  | Ex3R | GGG GAC AGC ATC AAA TCA TCC | 12,064-12,044 |  |
| 4 | 403 | GE4F | TCC TCT GAC TGC TCT TTT C | 11,990-12,008 | **E4** 12021-12299 |
|  |  | GE4R | CTA AGG GTG AAG AGG AAT C | 12,401-12,383 |  |
| 5 | 399 | GE5F | TGC CGT GTT CCA GTT GCT TT | 12,982-13,001 | **E5** 13055-13238 |
|  |  | GE5R | TCC AAA TAC TCC ACA CGC AA | 13,380-13,361 |  |
| 6 | 262 | GE6F | CAG ATA GCG ATG GTG AGC AG | 13227-13246 | **E6** 13320-13432 |
|  |  | GE6R | GCC ACT GAC AAC CAC CCT TA | 13488-13469 |  |
| 7 | 225 | GE7F | TGC CAC AGG TCT CCC CAA GG | 13943-13962 | **E7** 14000-14109 |
|  |  | GE7R | AGG GGT CAG CGG CAA GCA GA | 14177-14158 |  |
| 8 | 269 | GE8F | ACC TGA TTT CCT TAC TGC CT | 14404-14423 | **E8** 14452-14588 |
|  |  | GE8R | GAG GCA AGG AAA GGT GAT AA | 14672-14653 |  |
| 9 | 228 | GE9F | GTA AGC AAG CAG GAC AAG AA | 14589-14608 | **E9** 14681-14754 |
|  |  | GE9R | ACG GCA TTT TGA GTG TTA GA | 14816-14797 |  |
| 10 | 215 | GE10F | CTT CTC CCC CTC CTC TGT T | 17546-17564 | **E10** 17572-17678 |
|  |  | GE10R | GGC AGG ATG AGA ATG GAA T | 17760-17742 |  |
| 11 | 211 | p53 × 11F | AAA GCA TTG GTC AGG GAA AAG | 18518-18538 | **E11** 18599*18680-19876 |
|  |  | p53 × 11R | AGA GAT GGG GGT GGG AGG CTG TC | 18728-18706 |  |
|  |  |  |  | GenBank: X54156 |  |
