# Supplementary material for: Effects of MDM2, MDM4 and TP53 Codon 72 Polymorphisms on Cancer Risk in a Cohort Study of Carriers of TP53 Germline Mutations
Source: PLoS One. 2010 May 26;5(5):e10813. doi: 10.1371/journal.pone.0010813 (PMC2877078; doi:10.1371/journal.pone.0010813)
Supplement: Table S2 — Primer sequences for genotyping assays. (0.03 MB DOC) [file pone.0010813.s011.doc]

|  | Assay | |
| --- | --- | --- |
|  | ***TP53 R72P*** | ***MDM2 SNP309*** |
| Forward PCR Primer(5′-3′)* | *GACGGGACACCGCTGATCGTTTA*CACTGAAGACCCAGGTCCAGAT | GGGAGTTCAGGGTAAAGGTCACG |
| Reverse PCR Primer(5′-3′)* | CCGGTGTAGGAGCTGCTGG | *GACGGGACACCGCTGATCGTTTA*TAGTGACCCGACAGGCACCT |
| Pyrosequencing Primer(5′-3′) | GGTGCAGGGGCCACG(reverse direction) | GGGCTGCGGGGCCGCT(forward direction) |
| Sequence to Analyze | **G/C**GGGGAGCAG | **G/T**CGGCGC |
| SNP | G/C | G/T |
| Amplicon Size(bp) | 109 | 116 |
| Annealing Temperature(^o^C) | 58 | 54 |

*The biotinylated universal primer (5′-3′) was Biotin-GGGACACCGCTGATCGTTTA; the universal sequence is underlined in the tails of the gene-specific primers.
