# Supplementary material for: Effects of MDM2, MDM4 and TP53 Codon 72 Polymorphisms on Cancer Risk in a Cohort Study of Carriers of TP53 Germline Mutations
Source: PLoS One. 2010 May 26;5(5):e10813. doi: 10.1371/journal.pone.0010813 (PMC2877078; doi:10.1371/journal.pone.0010813)
Supplement: Table S3 — Univarible and multivariable analyses of MDM2, MDM4, and p53 codon 72 polymorphisms on age of tumor diagnosis using raw genotype data from carriers of a p53 germline mutation. (0.04 MB DOC) [file pone.0010813.s012.doc]

| **Model** | **Polymorphism** | **Subcategory** | **Hazard Ratio** | ***P*-value** |
| --- | --- | --- | --- | --- |
| Univariable Analysis | *MDM2* | GG/GT | 1.21(0.74-1.97) | 0.4495 |
|  |  | TT | 1.00 |  |
|  | *MDM4* | GG/AG | 1.27(0.78-2.04) | 0.3111 |
|  |  | AA | 1.00 |  |
|  | *p53* codon 72 | PP | 2.18(1.26-3.79) | 0.0056 |
|  |  | PR/RR | 1.00 |  |
| Multivariable Analysis | *MDM2* | GG/GT | 1.23(0.74-2.04) | 0.4287 |
|  |  | TT | 1.00 |  |
|  | *MDM4* | GG/AG | 1.92(0.91-4.06) | 0.0872 |
|  |  | AA | 1.00 |  |
|  | *p53* codon 72 | PP | 2.05(0.98-4.31) | 0.0575 |
|  |  | RR/PR | 1.00 |  |
