# Supplementary material for: Effects of MDM2, MDM4 and TP53 Codon 72 Polymorphisms on Cancer Risk in a Cohort Study of Carriers of TP53 Germline Mutations
Source: PLoS One. 2010 May 26;5(5):e10813. doi: 10.1371/journal.pone.0010813 (PMC2877078; doi:10.1371/journal.pone.0010813)
Supplement: Table S4 — Distribution of allele frequencies by ethnicity. (0.03 MB DOC) [file pone.0010813.s013.doc]

| **Polymorphism** | **Allele** | **White** | **Black** | **Others** | ***P*-value*** |
| --- | --- | --- | --- | --- | --- |
| *MDM2* SNP309(n=264) | G | 74(41.6) | 3(8.8) | 16(30.8) | 0.0009 |
|  | T | 104(58.4) | 31(91.2) | 36(69.2) |  |
| *MDM4* (n=260) | A | 52(28.9) | 25(89.3) | 22(42.3) | <0.0001 |
|  | G | 128(71.1) | 3(10.7) | 30(57.7) |  |
| *p53* codon 72(n=264) | P | 37(20.8) | 19(55.9) | 12(23.1) | <0.0001 |
|  | R | 141(79.2) | 15(44.1) | 40(76.9) |  |

* Chi-square test
