# Supplementary material for: Effects of MDM2, MDM4 and TP53 Codon 72 Polymorphisms on Cancer Risk in a Cohort Study of Carriers of TP53 Germline Mutations
Source: PLoS One. 2010 May 26;5(5):e10813. doi: 10.1371/journal.pone.0010813 (PMC2877078; doi:10.1371/journal.pone.0010813)
Supplement: Table S5 — Genetic model selection using AIC in univariable analysis of MDM2, MDM4, and p53 codon 72 polymorphisms on age of tumor diagnosis using raw plus imputed genotype data among carriers of a p53 germline mutation. (0.05 MB DOC) [file pone.0010813.s014.doc]

| **Polymorphism** | **Subcategory** | **Hazard Ratio** | **Pr>ChiSq** | ***P*-value** | **-2LogL** | **AIC** |
| --- | --- | --- | --- | --- | --- | --- |
| *MDM2*(n=175) | GG | 1.43(0.87-2.35) | 0.1518 | 0.2017 | 1208.611 | 1212.611 |
|  | GT | 1.46(0.92-2.32) | 0.1099 |
| *p53* codon 72(n=174) | PP | **2.38(1.35-4.19)** | 0.0028 | 0.0102 | 1198.439 | 1202.439 |
|  | PR | 1.17(0.89-1.54) | 0.2532 |
| *MDM4*(n=174) | AG | 1.44(0.86-2.41) | 0.1698 | 0.2682 | 1199.815 | 1203.815 |
|  | GG | 1.43(0.90-2.27) | 0.1282 |
| *MDM2* (G dominant) | GG/GT=1,TT=0 | 1.45(0.96-2.19) |  | 0.0764 | 1208.617 | **1210.617** |
| *MDM2* (G recessive) | GG=1, GT/TT=0 | 1.21(0.78-1.89) |  | 0.4020 | 1212.105 | 1214.105 |
| *MDM2* (G additive) | GG=2,GT=1,TT=0 | 1.24(0.97-1.59) |  | 0.0923 | 1209.637 | 1211.637 |
| *p53* codon 72(P dominant ) | PP/PR=1,RR=0 | 1.26(0.96-1.65) |  | 0.098 | 1200.902 | 1202.902 |
| *p53* codon 72(P recessive) | PP=1,PR/RR=0 | **2.22(1.27-3.89)** |  | 0.0052 | 1199.094 | **1201.094** |
| *p53* codon 72(P additive) | PP=2,PR=1,RR=0 | 1.32(1.04-1.67) |  | 0.0218 | 1199.544 | 1201.544 |
| *MDM4*(G dominant) | AG/GG=1,AA=0 | 1.43(0.93-2.22) |  | 0.1054 | 1199.816 | **1201.816** |
| *MDM4*(G recessive) | GG=1, AG/AA=0 | 1.15(0.78-1.68) |  | 0.4873 | 1201.805 | 1203.805 |
| *MDM4*(G additive) | GG =2,AG=1,AA=0 | 1.16(0.91-1.48) |  | 0.2182 | 1200.661 | 1202.661 |
