# Supplementary material for: Effects of MDM2, MDM4 and TP53 Codon 72 Polymorphisms on Cancer Risk in a Cohort Study of Carriers of TP53 Germline Mutations
Source: PLoS One. 2010 May 26;5(5):e10813. doi: 10.1371/journal.pone.0010813 (PMC2877078; doi:10.1371/journal.pone.0010813)
Supplement: Table S6 — Multivariable analysis of hazard ratio for MDM2, MDM4, and p53 codon 72 polymorphisms on age of tumor diagnosis among carriers of a p53 germline mutation, probands excluded (n = 126). (0.04 MB DOC) [file pone.0010813.s015.doc]

|  |  | **All** (n=126)* | | **Male** (n=63)** | | **Female (**n=63**)**** | |
| --- | --- | --- | --- | --- | --- | --- | --- |
| **Polymorphism** | **Subcategory** | **Hazard Ratio** | ***P*-value** | **Hazard Ratio** | ***P*-value** | **Hazard Ratio** | ***P*-value** |
| *MDM2* | GG/GT | **1.79(1.14-2.81)** | **0.0117** | 1.67(0.86-3.27) | 0.1315 | **1.90(1.18-3.08)** | **0.0089** |
|  | TT | 1.00 |  | 1.00 |  | 1.00 |  |
| *MDM4* | AG/GG | 1.71(0.95-3.09) | 0.0752 | 1.38(0.76-2.52) | 0.2891 | 1.97(0.88-4.43) | 0.0998 |
|  | AA | 1.00 |  | 1.00 |  | 1.00 |  |
| *p53* codon 72 | PP | **8.59(5.24-14.07)** | **<0.0001** | **6.97(3.24-15.02)** | **<0.0001** | **7.65(2.84-20.56)** | **<0.0001** |
|  | PR/RR | 1.00 |  | 1.00 |  | 1.00 |  |

*Adjusted for sex, race, and birth year

**Adjusted for race and birth year
